# Lytic lesions: looking lethal but leaving room for a simple cure? A case of *Veillonella* spinal osteomyelitis

**DOI:** 10.1099/jmmcr.0.005108

**Published:** 2017-09-01

**Authors:** Sarah Baker, Rebecca Allyn

**Affiliations:** ^1^​ School of Medicine, University of Colorado, 13001 E. 17th Place, Aurora, CO 80045, USA; ^2^​ Denver Health Medical Center, 777 Bannock Street, Denver, CO 80204, USA

**Keywords:** osteomyelitis, *Veillonella* bacteraemia, lytic lesions, back pain, ceftriaxone

## Abstract

**Introduction.** Diagnosing clinically significant infection caused by *Veillonella* species can be a challenge. *Veillonella* species are usually found in polymicrobial processes and are often regarded as a contaminant. Additionally, they are slow to grow in culture and this can lead to a delay in diagnosis or a missed diagnosis. *Veillonella* species rarely cause serious infections, but have been found to cause bacteraemia and osteomyelitis.

**Case presentation.** A 67-year-old man with a history of treated prostate cancer presented with 2 weeks of progressive lower back pain and weakness. He had no signs or symptoms of active infection. He was found to have multiple lytic lesions in his lumbar spine that were initially suspected to be secondary to metastatic cancer. However, tissue and blood cultures were ultimately consistent with infection by *Veillonella* species.

**Conclusion.** This case report highlights the fact that uncommon illnesses can often present like common disease processes. Because of the radiological appearance of the patient’s lesions and his lack of infectious symptoms, a diagnosis of metastatic cancer was initially thought to be likely. Relying on the pathology and culture data, and waiting on the initiation of antimicrobials until the diagnosis was accurately established, were important factors in diagnosing and treating this infection. *Veillonella* species can be true pathogens when found in isolation and associated with bacteraemia. Additionally, they can cause an indolent infection that can lead to osteomyelitis. Failure to accurately diagnose this infection in a timely manner would have led to ongoing debility and diagnostic uncertainty for this patient.

## Abbreviations

MRI, magnetic resonance imaging; PSA, prostate specific antigen.

## Introduction

Uncommon disease entities can often present as common illnesses. Metastatic spinal lesions are common, while slowly progressive spinal osteomyelitis from *Veillonella* species infection is very rare. *Veillonella* is an anaerobic, Gram-negative coccus that forms part of the normal microflora in the oral cavity, upper respiratory tract, gastrointestinal tract and vagina. *Veillonella* species are usually found in polymicrobial processes. Isolated *Veillonella* infection is rare, but has been found to cause dental infections, pulmonary infections, meningitis, endocarditis, osteomyelitis and prosthetic joint infections [1]. *Veillonella* *parvula* is the most common virulent species. There have been two reports of *Veillonella* foot osteomyelitis in diabetics, seven reports of vertebral osteomyelitis [2] and one report of osteomyelitis after an open fracture to the left radial bone. The infection has also been associated with endoscopic biopsies, open fractures, post-procedural oesophageal perforation, rheumatoid arthritis in the setting of immunosuppression and possibly poor dentition [1, 3]. Some other reported cases did not find an underlying source or risk factor. Some patients presented with signs of infections, while others only had sub-acute pain. Bacteraemia was common in these case reports.

## Case report

**Fig. 1.**
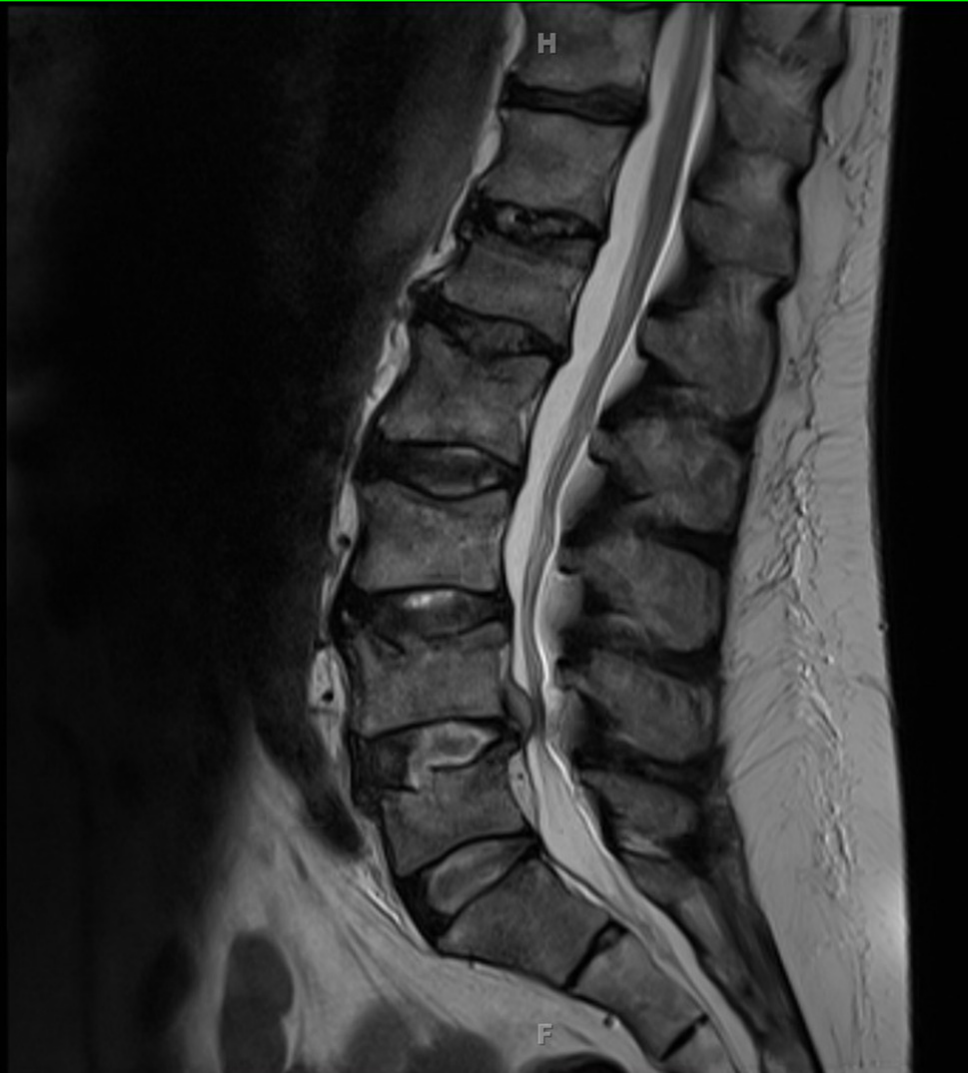
Lumbar MRI. MRI scan showing minimal to mild superior compression fractures from L1 through L5 with variable areas of enhancing oedema, with small lytic lesions throughout the lumbar spine and a large lytic lesion at the L5 vertebral body.

A 67-year-old man with a history of treated prostate cancer, alcohol abuse and alcoholic cirrhosis presented to an urban, academic hospital with new low back pain. The pain had started 2 weeks prior to his presentation, had gradually worsened, and then became associated with bilateral lower extremity shooting pain and weakness. He denied bowel or bladder incontinence or saddle anaesthesia. He also denied subjective fevers or chills or any other bothersome symptoms. He was found to have normal vital signs, poor dental hygiene, tenderness over the lumbar spinous processes, and normal neurological and prostate exams. Magnetic resonance imaging (MRI) of the spine (Fig. 1) showed mild compression fractures, with small lytic lesions throughout the thoracic and lumbar spine, and a large lytic lesion at the L5 vertebral body. The patient had a normal prostate specific antigen (PSA) level and a normal white blood cell count. C-reactive protein was mildly elevated at 30 mg l^−1^ (normal range 0–10 mg l^−1^).

There was concern for metastatic cancer given the patient's prior history of prostate cancer. His normal PSA level was not reassuring, because his prior prostate cancer had been diagnosed in the setting of a normal PSA level. He was started on intravenous dexamethasone 4 mg every 6 hours given the concern for metastatic spinal lesions and a neurosurgical consult was obtained. The neurosurgery team did not feel that urgent surgical intervention was needed and thus a biopsy of the lesion was obtained by the interventional radiology team for diagnostic purposes. Surprisingly, the vertebral biopsy result showed no evidence of malignancy, but did show findings consistent with osteomyelitis and was sent for culture. Dexamethasone was discontinued and blood cultures were drawn. Antibiotic therapy was withheld while culture data was pending, as the patient was clinically stable.

The patient was observed while the cultures were pending, and 2 days later *Veillonella* species started growing from the broth of the tissue biopsy and from one of two blood cultures. After a great deal of prompting for additional history, the patient reported a mechanical fall 4 weeks prior to the onset of his pain, at which time several teeth had been dislodged. A trans-thoracic echocardiogram obtained to evaluate for possible endocarditis was negative. Additionally, repeat blood cultures obtained to ensure clearance of the bacteraemia after the initiation of antibiotics were also negative. Culture sensitivity results were obtained, but were delayed as the sample had to be sent out to a reference laboratory for further testing. In the meantime, ceftriaxone 1 g intravenous daily was started and the patient was discharged to a nursing home for further rehabilitation, with close clinical follow-up scheduled. The sensitivity testing was performed by broth microdilution and showed that there was no β-lactamase inhibition. The MIC results showed sensitivity to meropenem, ampicillin/sulbactam and piperacillin/tazobactam, and borderline sensitivity to metronidazole and penicillin. The results were delivered with the caveat that there are currently no Clinical and Laboratory Standards Institute guidelines in the USA for the performance and interpretation of susceptibility testing for anaerobes other than *Bacteroides fragilis*. Thus, the advice of the reference laboratory was to interpret these results with caution. At his follow-up appointment, the patient appeared to be having a good clinical response to ceftriaxone. Given the lack of β-lactamase inhibition in the culture data and the fact that once daily dosing of antibiotics decreases disruption of therapies, ceftriaxone was continued. The patient completed a 6 week course of antibiotics and had complete resolution of his symptoms.

## Discussion

In our case, it is thought that the primary source of *Veillonella* osteomyelitis was the oral cavity. It is likely that the combination of poor dental hygiene and dental trauma during the patient's fall led to transient bacteraemia, which seeded his vertebrae. There was a delay in the diagnosis because the patient did not have overt signs or symptoms of infection. Additionally, given his prior history of cancer and the appearance of the lesions on his imaging scans, it was thought that the patient very likely had a new metastatic cancer. The details about the dental trauma did not come to light until it was becoming increasingly apparent from the culture data that the patient had osteomyelitis and he was specifically quizzed about this detail. Additionally, because the *Veillonella* species was initially growing only from the broth of the tissue biopsy, there was concern that this represented a contaminant. While broth cultures enhance sensitivity in detecting slow growing or fastidious bacteria, they also yield more growth of contaminant bacteria. The diagnosis was confirmed by correlating the clinical scenario with bone biopsy and blood culture data.

*Veillonella* species rarely cause infection; however, an indolent infection can lead to devastating lesions. *Veillonella* species are usually recovered in polymicrobial processes and are often regarded as a contaminant. However, *Veillonella* can contribute to pathogenic polymicrobial processes and, less commonly, can cause infections in isolation [4]. Risk factors for *Veillonella* infections appear to be immunosuppression, diabetes, malignancy, collagen disease, periodontal disease, open fractures and instrumentation with endoscopy. However, *Veillonella* infection can also occur in healthy individuals without any risk factors. The most common types of infection are bone and joint infections, followed by endocarditis and bacteraemia [4].

There have been two reports of *Veillonella* foot osteomyelitis in diabetics, seven reports of vertebral osteomyelitis [2] and one report of osteomyelitis after an open fracture to the left radial bone [4]. One case of thoracic osteomyelitis occurred in a 74-year-old male with poor dental hygiene who presented with only thoracic back pain and was found to have *Veillonella* in a bone biopsy culture. Blood cultures were either negative or not obtained [5]. It was thought that the patient had haematogenous spread from periodontal disease or intestinal translocation. In two other cases, diabetic patients presented with septicaemia and osteomyelitis of the foot. Both blood and tissue cultures were positive for *Veillonella* [1, 6]. In another case, a 31-year-old man developed a fever after a cervical fusion and was found to have *Veillonella* osteomyelitis [7]. No blood cultures were obtained. The possible source of entry of the organism was thought to be an oesophageal perforation that had occurred during surgery. Another case reported a 61-year-old female who was on chronic prednisone for rheumatoid arthritis and Sjogren’s disease, who presented with low back pain and fever. She was found to have lumbar osteomyelitis and *V. parvula* growing in blood and tissue cultures [3]. Another case involved a 55-year-old man who presented with severe back pain and fever after undergoing a biopsy of the small intestine and rectum. He was found to have lumbar discitis and *V. parvula* in blood and tissue cultures [8]. There have also been two case reports from healthy patients who presented with only lower back pain and were found to have discitis. Tissue cultures were positive for *V. parvula* in both cases with blood cultures positive in one of these cases. The source of infection was unclear [1, 9].

*Veillonella* is usually vancomycin, tetracycline, aminoglycoside and ciprofloxacin resistant, and typically responds well to penicillin therapy [2]. *In vitro*, *Veillonella* is susceptible to cephalosporins, ceftriaxone, clindamycin, metronidazole and chloramphenicol. Currently, there are no clear treatment recommendations in the literature due to the small number of case reports on *Veillonella* as a pathogen. In some cases, *Veillonella* have shown resistance to penicillin [4]. The reported cases have used penicillin, imipenem, ceftriaxone and cefotaxime/metronidazole for treatment.

There has been an array of patient presentations with *Veillonella* osteomyelitis. Some patients have presented with sepsis and some with only back pain. Some have had obvious risk factors and some have been healthy. Most of the cases have found *Veillonella* in blood and tissue cultures. However, some have isolated the organism from only one source [2]. Improper sample collection and transport can cause failure of a strictly anaerobic organism to grow. Additionally, diagnosis can be delayed due to the slow growing nature of *Veillonella* (4–5 days). Also, sometimes infection may not initially be suspected because patients can present with non-specific symptoms. Further complicating diagnosis is the fact that *Veillonella* is often considered a contaminant when it does appear in a culture. The key to the correct diagnosis is patience, along with careful correlation of the clinical scenario, biopsy data and culture data from blood and tissue sources.
